# Theorising health professionals’ prevention and management practices with children and young people experiencing self‐harm: a qualitative hospital‐based case study

**DOI:** 10.1111/1467-9566.13211

**Published:** 2020-10-28

**Authors:** Sarah MacDonald, Catherine Sampson, Lucy Biddle, Sung Yeon Kwak, Jonathan Scourfield, Rhiannon Evans

**Affiliations:** ^1^ Centre for the Development and Evaluation of Complex Public Health Interventions for Public Health Improvement (DECIPHer) Cardiff University Cardiff Wales UK; ^2^ Population Health Sciences Bristol Medical School University of Bristol Bristol UK; ^3^ School of Medicine Cardiff University Cardiff UK

**Keywords:** Mental health and illness, self‐harm, suicide, interviews, doctor–patient communication/interaction

## Abstract

Self‐harm in young people remains a significant concern. Studies of emergency departments have centred on negative professional attitudes. There has been limited interrogation and theorisation of what drives such attitudes, and the contexts that sustain them. Adopting a complex systems lens, this study aimed to explore how systems shape professional and patient interactions. It draws upon interviews with healthcare and affiliated professionals (*n* = 14) in a UK case study hospital, with primary focus on the emergency department. Data were analysed using a thematic approach and the principles of grounded theory. Four themes emerged, with the first three centralising how professionals’ practices operate within: (1) a framework of risk management; (2) expectations of progressing patients through the care pathway; and (3) a culture of specialist expertise, with resulting uncertainty about who is responsible for self‐harm. The fourth theme considers barriers to system change. A small number of participants described efforts to enact positive modifications to practices, but these were frustrated by entrenched system structures. The potential detrimental impacts for patient care and professional wellbeing are considered. Future practice needs systemic action to support professionals in treating patients experiencing self‐harm, while future research requires more ethnographic explorations of the complex system in situ.

## 1 Introduction

Self‐harm, which may be defined as the internal or external infliction of damage to the body, remains a significant concern in relation to children and young people (Doyle *et al*. 2015, Hawton *et al*. 2012, Morgan *et al*. 2017, Muehlenkamp *et al*. 2012). Despite uncertainty about the extent of increases in prevalence rates (Doyle *et al*. 2015, Morey *et al*. 2016, Muehlenkamp *et al*. 2012), there are clearly growing numbers of young people attending emergency departments for professional treatment and care. Clinical guidance on the short‐term prevention and management of self‐harm has been issued in the UK to ensure high quality care (National Institute for Health and Care Excellence 2004). This prescribes that following presentation to an emergency department, an individual should receive medical or surgical treatment and a psychosocial assessment, to be carried out by specialist mental health professionals, such as CAMHS professionals (Child and Adolescent Mental Health Service) in the case of children and adolescents. Assessment informs care planning, which includes the decision to admit an individual or discharge them to the support of community services.

Despite clear NICE guidance, there is evidence of variable implementation (Cooper *et al*. 2013). Equally, patient experiences report that seeking help, or in some cases being coerced to receive help, within an emergency hospital setting can be difficult. Studies describe how those who self‐harm have been unhelpfully characterised, with patients being the subject of negative attitudes and punitive treatment (Chandler 2016, Gibb *et al*. 2010, Hadfield *et al*. 2009, Owens *et al*. 2016, Saunders *et al*. 2012). A recent systematic review of patient experiences of presenting to emergency departments following self‐harm reported variability in care quality, while also revealing the mechanisms through which negative experiences may contribute to further self‐harm and reduced disclosure or help‐seeking in future (MacDonald *et al*. 2020). For young people, there are mixed experiences of care quality, including a degree of sympathy and compassion from professionals in addition to reports of feeling ignored and experiencing disorientation when transitioning into and out of care (Gulliver *et al*. 2010).

Recommendations to remedy inadequacies in self‐harm provision have tended to focus on individual‐level change including improvement to clinical training in order to enhance knowledge, skills and confidence (Catledge *et al*. 2012, Saunders *et al*. 2012). Yet, there remains uncertainty as to whether such approaches address the determinants of professional practices, primarily because there has been limited empirical consideration of the key drivers of professional and patient interactions, and the structural context that sustains them. Such attention to how hospitals institutionally determine care provision will also go some way in explaining variability in guidance implementation. The present paper seeks to address this extant research gap, adopting a complex system lens (Hawe *et al*. 2009, Moore *et al*. 2019, Salway and Green 2017) to understand how dynamics and interdependencies within a health system structure the everyday practice of managing and preventing self‐harm among presenting children and young people.

Conceptualising systems is difficult as it is a rather amorphous construct with early systems theorists highlighting the importance of networks of interactions between system actors and sub‐systems (Luhmann 1986, Parsons 1951). Sociological theorising has focused on the system’s identity as being (re) produced through communication over what is considered meaningful and what is deemed to be more inconsequential, and there is further consideration of embedded and interdependent systems (Luhmann 1986). This implies that rather than treating the hospital as a single entity, we see embedded systems across the care pathway, which has implications for role boundaries and areas of clinical expertise. Within the field of maternal health, researchers have highlighted how challenges to expertise are often met with defensiveness, with professionals retreating towards their dominant specialism (Hunter and Segrott 2014; Larson 1990). Similar insights are needed about professional role boundaries in relation to self‐harm, with a consideration of the implications of care pathways, which encourage inter‐professional collaboration.

A further relevant aspect of systems theory is the interest in relationships with broader sub‐systems including political, societal and cultural sub‐systems (Luhmann 1986). This echoes ongoing debates within the sociology of risk where commentators have highlighted the importance of framing risk within a complex set of wider discourses, recognising that decision‐making involves juggling risks alongside different sets of interests, values and priorities (Alaszewski 2009, Green 2009, Zinn 2009).

Drawing on interview data with healthcare and affiliated professionals, primarily working in the emergency department, this study aimed to explore accounts of service provision following a child or young person’s presentation to hospital for self‐harm, with or without a specified suicidal intent. It seeks to conceptualise the perceived system cultures and priorities that are inextricable from health professionals’ self‐harm management and prevention practices, both within the immediate hospital setting and wider healthcare system. In attempting to understand the system more wholly, while acknowledging it cannot be known in completeness, we can start to identify the determinants of care quality that have most leverage and are most malleable to modification in the attempt to enhance patients’ experiences.

## 2 Research methods

### 2.1 Research design

An instrumental case study approach provided in‐depth insights into a complex healthcare system in the UK, generating theory to understand the contextual determinants of professional practices (Crowe *et al*. 2011, Prato *et al*. 2019, Stake 1995). Combining case study methodology with a complex systems perspective has been advocated for studying complex adaptive systems, as it is useful for revealing unpredictable relationships and interactions within the system (Anderson *et al*. 2005, Moore *et al*. 2019).

### 2.2 Defining the case

The case study was one large hospital within a major UK urban centre. The hospital is part of a healthcare system governed by the local health authority, which also encompasses primary and community care, in addition to social and educational provision for children and young people. Within the hospital’s emergency department, treatment is sub‐divided, with paediatric emergency care treating children and young people up to the age of 16, and adult emergency care treating those aged 16 and over. On presentation for self‐harm, children and young people receive medical treatment and a psychosocial assessment, in accordance with NICE guidelines (National Institute for Health and Care Excellence 2004). Assessments are conducted by the local community‐based CAMHS Crisis Team consisting of mental health nurses and on‐call CAMHS psychiatrists (based in different geographical locations) when out of hours. Assessments are conducted in the emergency department or on the paediatric ward if a child or young person up to 16 years has been admitted directly to the ward. It is rare for a young person aged 16–18 to be admitted, with in‐patient provision generally provided at another location on an adult ward. CAMHS conduct follow‐up contact with the family within 1 month of the assessment.

### 2.3 Sample and recruitment

We recruited healthcare and affiliated professionals responsible for service delivery to children and young people presenting to the hospital with self‐harm. On commencement of the study, professional stakeholders were mapped across the system by the study team and collaborators (including professionals with experience of working at the site). Participants were purposively sampled and recruited through a range of strategies, including presentations at clinical team meetings. Snowball sampling was undertaken where initial interviewees identified other relevant professionals.

In total, the final sample included 14 participants, of which twelve were female. Table 1 presents an overview of participant characteristics. Further demographic details are not presented to ensure participant anonymity. Recruitment ceased when a range of participants from across the system had been interviewed and rich, representative data had been generated (Charmaz 2006, Saunders *et al*. 2018).

**Table 1:** Participants’ setting and role

Study ID	Setting	Role
Professional 1	Paediatric ward	Nurse
Professional 2	Paediatric emergency department	Doctor
Professional 3	Paediatric ward	Nurse
Professional 4	Voluntary support project	Project coordinator
Professional 5	Paediatric mental health, community	Nurse
Professional 6	Paediatric emergency department	Doctor
Professional 7	Paediatric emergency department	Doctor
Professional 8	Paediatric emergency department	Nurse
Professional 9	Paediatric emergency department	Nurse
Professional 10	Paediatric emergency department	Doctor
Professional 11	Adult emergency department	Nurse
Professional 12	Paediatric, community	Doctor
Professional 13	Paediatric mental health, community	Doctor
Professional 14	Paediatric mental health, community	Doctor

### 2.4 Method

In‐depth, semi‐structured interviews were conducted by one member of the research team. A topic guide addressed the following: professional and personal experiences of service delivery; system influences on service provision and practices; recommendations for service improvement; and opportunities to reduce future presentation for self‐harm. The aim was to examine experiences and encounters in detail and in context, and although the utilisation of interview data to explore experiences has been debated (Hammersley 2003, Willis 2019), the interactional data between interviewee and interviewer were useful in revealing an additional layer of meaning. At one level, data revealed their attitudes and experiences of the phenomena explored. At another level, the performance, construction and negotiation of professional identity in situ unveiled important insights into contested notions of expertise, knowledge and professionalism in relation to self‐harm (Evans 2018, Hunter and Segrott 2014). Interviews were conducted in the participant’s workplace and were audio‐recorded. Recordings were transcribed verbatim by a professional transcription service specialising in sensitive research areas. Interviews lasted between 24 and 63 minutes and were conducted between September 2018 and March 2019.

### 2.5 Ethical procedures

Participants were provided with study information in advance of interview, and queries were discussed prior to obtaining informed consent. While evidence reports that discussion of self‐harm for research purposes does not confer significant harm or distress (Blades *et al*. 2018), participants were provided with a list of support resources for follow‐up if required. Support for the primary researcher was obtained through routine supervision with an academic colleague. Ethical approval was provided by the NHS Research Ethics Committee to ensure the project was conducted in accordance with National Research Ethics Services (NRES) Standard Operating Procedures and the Governance Arrangements for Research Ethics Committees (GafREC) (Ref: 18/WA/0066).

### 2.6 Analysis

A thematic analytical approach was applied, with the principles of grounded theory in mind (Strauss and Corbin 1990). A coding framework was developed by indexing a subset of transcripts through ‘open’ coding and listening to recordings. The framework was confirmed before being applied to the remaining corpus of data and refinements were made where necessary. A subset of coded data was verified by a second researcher, with discrepancies resolved through discussion. Memos were recorded to capture changing researcher interactions with the data and also as a means of adding a complex systems lens, continually probing for reflections of the resources and priorities of the context (Birks *et al*. 2008).

Analysis progressed using tenets of axial coding to create categories that mapped across four constituent elements. First, we categorised codes according to the phenomenon under study, which we defined as professional and patient interactions where care was delivered. Second, we examined the strategies and actions that professionals used (e.g. the communication strategies). Third, we considered the wider conditions that give rise to the nature of the interaction and care (e.g. discourses of self‐harm). Fourth, we identified potential consequences for the professional and the patient (e.g. professionals’ emotional distress). Emergent themes were refined, expanded or collapsed through comparison both within and across categories. Four over‐arching meta‐themes were constructed that appropriately represent the data, and which serve as the basis of the present paper.

## 3 Findings

The findings present four overarching meta‐themes that explore health professionals’ experiences of interacting with children and young people who have presented to the hospital for self‐harm, and the central system drivers of practices within this context. The first three themes emphasise the influence of structural dynamics: (1) professionals’ construction of presenting individuals as ‘risks’ rather than ‘patients’, reflecting inscribed institutional discourses pertaining to risk management; (2) professionals’ stress on progressing patients through the care pathway, resonating with a wider culture of prioritisation of patient progress; and (3) professionals’ understanding of their bounded and discrete knowledge, linking to a training model that constructs professionals as specialists rather than generalists. The fourth theme reflects barriers to changing the system and how a small number of individual efforts to enact positive modifications to practices were frustrated by entrenched system structures.

## 4 Constructing the ‘patient’: a culture of risk and risk management

Professional accounts of treating and caring for children and young people presenting to the emergency department with experience of self‐harm were dominated by the notion of ‘risk’ which resonates with the pre‐occupation with risk within healthcare systems and the embeddedness of risk within everyday decision‐making (Hillman *et al*. 2013, Horlick‐Jones 2005). Positioning individuals within a nexus of risk rather than ‘illness’ meant that children and young people were sometimes seen as ineligible for medical treatment. Indeed, individuals were often differentiated from each other in terms of being ‘high risk’ or ‘low risk’, with the categorisation being used to pace progress through the care pathway and triage into an appropriate treatment plan. This process of categorisation is a reflection of system dynamics within the emergency setting, as caseloads are continuously fluctuating and patients seen as ineligible for treatment will absorb resources until there is adequate time and space to manage them (Buchbinder 2017). One professional described a specific ‘mental health room’ within the adult emergency department, which was designated for ‘high risk’ cases:

So they would be triaged, they would be, if they’re high risk to abscond, we have a specific ‐ it’s called a ‘mental health room’. There’s nothing in there, and there is an alarm all the way round. So if you’re in there, because some people are violent, some people are aggressive. (Professional 11, adult emergency department, nurse)

Pre‐occupation with risk, and the subsequent management of risk, was linked to the need to minimise the potential harm a child or young person may inflict upon themselves. From a systems perspective, it also reflected the institutional effort to balance the needs of numerous patients, rather than solely focusing on the requirements of the individual in question. Thus their response becomes shaped more by institutional and system expectations rather than responding directly to individual patient needs (Hillman *et al*. 2013). As such, risk assessments were often also underpinned by the rationale of minimising potential harm to others, especially where they were being admitted to a ward with multiple beds. One professional discussed working with staff from across the hospital to ensure that individuals with complex cases of self‐harm could be safely accommodated, as the disruptive behaviours linked to their case were a possible threat:

So it's those that we put the extra, definitely put the extra support in and then those that we know to be violent and aggressive to staff or other patients we've got to have a safe admission for those, so we’ll liaise with A&E… (Professional 1, paediatric ward, nurse)

The criteria for assessing risk, both to the individual and others, were complex and nebulous. Beyond the aforementioned display of ‘violent’ behaviour, one of the central considerations was the authenticity of the individual and their self‐harming practices, which added complexity in establishing the accuracy of risk (Chandler 2016, Scourfield *et al*. 2011). In one case, a clinician, who was based in community paediatrics, disassociated self‐harm as a medical issue and instead constructed self‐harm in relation to the young person’s social situation, constructing self‐harm as low risk:

They are perceived as at risk of doing something that could harm themselves and yet they didn’t have any medical diagnosis…They have attachment difficulties, usually the parents with mental health problems or personality disorder or drug misuse or are looked after children who are challenging because they’ve had an extremely disturbed early life. (Professional 12, paediatric, community, doctor)

Sub‐systems and structures were reflected in professionals’ constructions of self‐harm and the assignment of risk varied depending on professionals’ backgrounds and the settings in which they worked. Accounts from the voluntary sector participant who provided community services on discharge from hospital, constructed self‐harm as high risk where fears about ‘severe self‐harm’ or ‘accidental’ suicide were ‘always at the forefront of their minds,’ perhaps reflecting their own organisational ideology (Professional 4, voluntary support project, project coordinator). For those working in community paediatric mental health (CAMHS Crisis Team), there was a sense of weighing up risks in relation to a broader set of factors, influenced by their more extensive experience of self‐harm patients:

… you have to do risk assessment, you have to understand how severe, you have to understand how critical the family… So there’s lots of things that fit into the decision making regarding where things go. So it’s not cut and dry. So every case is individual and it’s not like it fits into everything. (Professional 13, paediatric mental health, community, doctor)

Participants working in this setting also spoke about their role in relation to supporting those working in the emergency department, where they helped medical colleagues, patients and their families manage risk:

Well the truth is, the truth is that a lot of our professional colleagues are very scared of psychiatric cases…they don’t understand the context to it… So a lot of times you’re reassuring medical staff, you’re reassuring patients, and patient families. (Professional 13, paediatric mental health, community, doctor)

Risk was further considered in relation to ‘more medically sick’ patients indicating implicit judgments about how treatment should be prioritised and allocated within the context of finite resources:

I suppose if someone comes in with superficial wounds during a busy shift, it’s … then you have got other more medically sick patients perhaps then it can be a bit. Or, annoying I guess is the right word, to have that in the mix and try and sort that out… (Professional 10, paediatric emergency department, doctor)

Patients’ condition was not absolute but rather a relative construct that informed decision‐making. Narratives were inscribed with reference to ‘others’ who were entitled care as they could not manage their own symptoms or negate the likelihood of a fatal outcome. In contrast, self‐harm was occasionally not deemed to qualify as a case of sickness that necessarily warranted intervention.

Professionals’ tendency to position individuals presenting with self‐harm within a nexus of risk and risk management had numerous implications for the quality and experience of care. In locating practices outside of the discourse of ‘sickness’, children and young people were sometimes seen as ineligible for medical treatment:

They’re incredibly needy and yet there is no medical solution for these kids and the only honest thing to say is, as a medic, this child has no medical condition… (Professional 12, paediatric, community, doctor)

Certainly, for some participants there was a sense that individuals who self‐harmed were responsible for knowing and mitigating their own risk, preferably at home, with professionals facilitating these practices:

Lots of people have had these self‐harm things for years and years and years, and they’ve managed them quite well at home, and by giving them the tools to be able to continue manage things at home. (Professional 11, adult emergency department, nurse)

One professional in particular drew parallels with presentations by first time mothers for failing to try out personal strategies to address the problem before seeking treatment for a non‐medical issue:

…probably would put it on par with being similar to, we see a lot of young babies. Sometimes first time mums or who haven’t got wider network of support of caring, who come in with like normal baby problems, and it’s those kind of things where sometimes you’re like, there are people here who are sick and you’ve come in and you didn’t try the other bits first. So it’s a bit of a frustration that you don’t maybe have a problem that needs to be here. (Professional 2, paediatric emergency department, doctor)

Where self‐harm was classified as being high risk, care was considered to move towards an approach that could at best be described as ‘defensive’ which conforms with broader tendencies towards self‐protection in response to institutional cultures of risk and blame (Hillman *et al*. 2013). Here, professionals described being motivated by a sense of fear at the potential consequences of their (in)action combined by the need to feel secure that they had taken every possible measure:

A lot of the time I sometimes think it is maybe a bit overkill but at the same time if God forbid something was to happen to that child next week at least I could say, ‘well I referred them’…In a way it's a really defensive way of working but at the same time if I didn't refer I know I wouldn't sleep at night, so that's how we work things. (Professional 9, paediatric emergency department, nurse)

The centrality of risk also had implications for health professionals themselves, with participants reflecting on the need for the system to provide more adequate support. As others have noted, positive system structures such as supervisions and opportunities for de‐briefing can provide important outlets for reflection and sharing experiences (O'Connor and Glover 2016). Primarily, this was because when a presentation of self‐harm was constructed as ‘risky’ it was loaded with significant and troubling connotations, which was difficult for a single clinician to manage alone. One professional commented on the need for peer support:

…up until that point there was like one person holding what might be a very risky case without necessarily having consultation supervision reflection. (Professional 5, paediatric mental health, nurse)

Without such support, a clinician’s wellbeing could be adversely impacted.

## 5 Progressing the ‘patient’: ‘Moving on’ through the system

Against a backdrop of needing to process patients through a care pathway, and with the particular emphasis on quick turnover within the emergency department, presentations of self‐harm were often considered disruptive system events with actors within the system working to restore order (Murphy *et al*. 2018). The emphasis on patient progress within the emergency department system and the way in which care pathways determine the flow and movement of the self‐harmed body reflects wider temporal constraints placed on health service delivery (McWade 2015, Soldatic 2013).

For many participants, presentations of self‐harm signalled a change in rhythm, a break in the traditional flow of triaging and a shift in the emotional expenditure required by professionals. The disruptiveness of these cases led to them being characterised as ‘heart sink presentations’, with one doctor working in emergency paediatrics reflecting on the protracted period of time and resource such a case would require:

Amongst my colleagues and health professionals as a whole I think the people think of it as a bit of a heart sink presentation with that, because you know that they’re going to be a patient that you’re going to take quite a long time with. (Professional 6, paediatric emergency department, doctor)

Such sentiments characterised a number of accounts. There were repeated experiences of feeling discouraged by having to treat complex cases within a busy and pressured environment, although any type of presentation could be troubling when it was late into a shift in the emergency department:

… I do sometimes get a heart sink when I see CAMHS patients come up on the board. It’s 10 o’clock and it’s really busy, but it’s no different to my heart sinking if there is, let’s say, an itchy ear that comes in at the same time. (Professional 10, paediatric emergency department, doctor)

Reflections on self‐harm cases included direct comparisons with other patients and conditions, and although such comparisons (such as an ‘itchy ear’) may seem illogical as they seem to be comparing different things, without further data that provide patient insights we do not know how these sorts of comparisons are interpreted and experienced.

The pressure of patient progress was most clearly brought into sharp relief in the case of frequent attenders, who are a particular concern given the increased risk of subsequent suicide (Chan *et al*. 2016). In some instances, the long shift work required in the emergency department meant that some professionals could see the same young person twice during the same shift. The sense of there being repeated emotions, processes and conversations left some participants feeling that they were fighting a losing a battle, with little hope of recovery:

…I’m constantly having the same conversations with you and you know, it’s a cry for help or a behavioural issue or whatever, but we’re not getting anywhere. (Professional 6, paediatric emergency department, doctor)

Such cases were often characterised by a sense of professionals’ frustration and failure. Perhaps even more evident was the sense of emotional energy expended. Indeed, there was recognition that participants would have to try harder to achieve progress for the patient, but it could be difficult to source motivation:

…but if you see a name pop up and you’re like ah, they’re again. It is quite difficult to kind of motivate yourself to … (Professional 6, paediatric emergency department, doctor)

While the incongruence between the system dynamics and the nature of self‐harm presentations was dispiriting for professionals, it also seemed linked to the quality of care they felt they provided. The culture of moving patients through the care pathway meant that there was at best fleeting interaction, and a real struggle to foster positive relationships. Moreover, participants spoke about the risks of seeking to build connections with presenting individuals. In particular, they spoke about the potential harm of encouraging individuals to share their history only to be moved on and not seen by that professional again:

I think that is the part of the sticking point between staff feeling like, I’d like to invest and look after you and have loads of empathy for you, but at the same time to do that, I kind of need to get to know you and you need to get to know me and share. But I don’t want to make you share, because I might not see you again. (Professional 2, paediatric emergency department, doctor)

Such data provided insight into the extent of conflict experienced by professionals when interacting with presenting individuals. They were torn between wanting to build rapport and show empathy, but at the same time recognising that building a relationship can be emotionally difficult and ultimately futile if their interaction is only a moment in the care pathway.

## 6 Treating the ‘patient’: Bounded expertise and expectations of the ‘other’

Beyond reflecting on the treatment of the individual presenting with self‐harm, participants also considered the professionals who they felt were best placed to provide care. Central to accounts was debate over where ‘expertise’ was located within the system, and if the professional in question could be defined as having expert knowledge. Other research notes that that this sort of boundary work or guarding the perimeters of emergency settings is perhaps less about determining if patients were deserving of care and more about determining the appropriateness of the emergency setting for patient care (Buchbinder 2017).

A number of participants maintained that they were in possession of professional expertise to treat self‐harm presentations; the requirements of presenting cases could be met by their skills. For example, one particular nurse, while acknowledging the challenges involved, felt equipped to deal with self‐harm as a result of their technical and experiential knowledge. This included overcoming fears about asking patients whether they held suicidal thoughts:

It's hard. It's not easy but with experience it's got to be asked, and I think once you’ve got over the fear that our nursing staff, and I know perhaps I would've had a long time ago, is you're planting that seed by asking, is once you’ve got over that and know that's not true, then you ask because it's the best way of keeping these young people safe. (Professional 1, paediatric ward, nurse)

In contrast, others struggled in treating cases of self‐harm, deeming it to be outside their specialist area, but within the purview of others within the system. Indeed, some participants spoke about the risk of compounding the problems of presenting individuals ‘seeing the wrong person’, implicitly suggesting that there was the other, ‘right’ person who would know what to do (Professional 2, paediatric emergency department, doctor). Across participants, nurses seemed more flexible in their approach, but attitudes also varied depending on the setting in which they worked. Accounts from a doctor working in community paediatrics gave a sense of not wanting to ‘mess things up’ or for self‐harm to escalate to suicidal intentions, and for that reason they held strong views about professional role boundaries, often keeping their distance and remaining ‘within a medical box’:

We need to know how not to mess things up in a disastrous way through our necessary contact with these patients. Our job is to help people and there’s a risk that we can make things worse if we’re not careful. That’s one of the reasons I would generally, although I regard myself as a fairly minded doctor, it’s one of those situations where I’d probably keep myself quite firmly within a medical box because I think somebody else needs to do it to have more continuity and more expertise than me. (Professional 12, paediatric, community, doctor)

Located between these two positions, was the general sense of uncertainty and ambiguity over participants’ expertise and role. One doctor located within the paediatric emergency department maintained that despite often being the first point of contact for self‐harm presentations, they felt that they did not have knowledge to differentiate between higher risk and lower risk cases:

…I think there’s a feeling of not knowing quite what your role is, and have been tasked with the person to say whether somebody is safe or not safe, that’s not necessarily feeling like you have had the training to do it… (Professional 2, paediatric emergency department, doctor)

Doubts over expertise had implications for the treatment of self‐harm presentations. It impacted on professionals’ confidence in interactions while the individual was in hospital. Moreover, the lack of knowledge about what might happen at the next point in the care pathway and beyond discharge left them unsure about the validity and appropriateness of any support, making them hesitant to offer advice:

You don’t necessarily have the answers. I feel quite sorry for them that quite often you don’t know where to signpost them onto and what to do next…you don’t want someone to present and then you just go ‘well, I’m sorry, but there’s nothing much I can do’. (Professional 6, paediatric emergency department, doctor)

Hence, we see professionals grappling with a dual issue. On the one hand, they understand that expertise is located in the system, even if it does not reside with them personally. On the other hand, the lack of certainty over where this expertise is on the care pathway, and who might embody it, makes them reticent to offer support, as they are unsure of the consequences of their actions.

## 7 Barriers to changing the complex system

Amidst challenges to handling self‐harm presentations, which were often linked to entrenched institutional practices and structures, a small number of participants described their personal efforts to act upon the system in the direction of positive change. These examples illustrate the way in which system agents respond to system inadequacies drawing on their expertise, experiences, values and institutional rules (Keshavarz *et al*. 2010).

Personal efforts to innovate included the introduction of small improvements in care quality, such as providing ‘a little adolescent DVD box’ in order to make children and young people feel more relaxed especially if they had a long wait, providing a distraction from current events. The same nurse also described making the hospital experience more amenable for carers by providing food, drinks and a listening ear:

…So you make sure they’ve got drinks, you make sure they're fed, you make sure that they're listened to when they need to be listened to and they’ve got, I don’t mind hugging a parent if they're crying, I have no problem with that. (Professional 8, paediatric emergency department, nurse)

Equally, there were some attempts to reduce the need for patients to ‘repeat the story over and over’ as a way of minimising emotional distress and making ‘the whole journey smoother’ (Professional 1, paediatric ward, nurse). There appeared to be recognition of the potential churn of emotions that patients might encounter as they move through the system and are subject to numerous structures of surveillance as they are constantly asked to repeat their stories and justify their presentation (Soldatic 2013). By trying to minimise patient distress, health professionals seemed to be showing sensitivity to the challenges and barriers patients encounter as they navigate the healthcare system (Buchbinder 2017).

Individual participants also sought to change sub‐systems and shift the culture around how self‐harm is perceived within the emergency department. To enable this, there was a keen focus on developing professional expertise around self‐harm. This included: undertaking academic studies related to self‐harm; developing links with support groups; joining national committees and steering groups; and networking with other NHS Trusts to exchange experiences. Efforts also extended to install support mechanisms for colleagues who were working in the complex system of risk and patient progress. Participants recognised that colleagues might be exposed to ‘hearing lots of sad things’ on an initial presentation of self‐harm, especially in cases where there was associated suicidal intent, but the rapid churn of activity ensured minimal time to process any potential distress. As such, there was individual advocacy for staff supervision but this seemed dependent on individuals providing and accessing support rather than embedded supportive systems:

…any nursing is stressful and you hear lots of sad things but when you're hearing lots of sad things and people wanting to die it's not a good thing to hear…I'm supported really well and I feel quite passionate that other people should be supported. So I often support staff on the ward and therefore I hold theirs and therefore I'm supported as well and that’s important. (Professional 1, paediatric ward, nurse)

Attempted changes were largely driven by individuals who were passionate about their role, and keen to correct the inadequacies in their immediate context, which prevented them from ‘doing a good job’ (Professional 2, paediatric emergency department, doctor). Yet efforts of individualised agency were limited against a backdrop of seemingly intractable system‐level structures, ways of working, and limitations of space and time. Where participants sought to improve the quality of interactions, they struggled to balance the needs of different patients:

…I mean we haven’t always got the time to chat with them really when you’ve got a couple of other patients demanding, sometimes it feels like we've not got the time to spend with them properly. (Professional 3, paediatric ward, nurse)

Equally, while participants sought to make small changes to let an individual know that someone cared for them, this was playing out within a context of ‘patient progress’, where presenting individuals were reduced to a number on a screen:

The patient who's waiting for CAMHS is sat in the corner of the waiting room it's very easy to be like ‘they don’t care about me; I'm literally a number on the screen.’ So that's why I always try and make eye contact with them, if I see them in the waiting room and I know that they're waiting, I'm like ‘I know you're there, I know and I'm going to change something now’. (Professional 9, paediatric emergency department, nurse)

Attempts to improve care were further halted by the inappropriateness of clinical space. The lack of privacy and the absence of a ‘quiet nurturing space’ made it difficult to think about building relationships to meet children and young people’s needs:

There’s no privacy, there’s no real quiet nurturing space where you can go and talk about significant emotional needs if that’s what you need to do. (Professional 12, paediatric, community, doctor)

Throughout accounts of participants acting to change the system, was acknowledgment of being bounded by their particular context. Indeed, they discussed the challenge of having impact beyond their discrete point in the care pathway, as it would require moving in to ‘a separate system’ that was currently beyond their reach:

…but I feel like we are out of the system, that is going to provide. We’re a separate system, to that’s going to provide the ongoing care, and therefore, we can’t, we’re not part of it and we can’t help it. (Professional 2, paediatric emergency department, doctor)

While a number of individuals were clearly driven by a passion to improve care quality, it is apparent that system‐level barriers often thwarted the exercise of agency, within the immediate but also within the interdependent systems considered to exist across the care pathway.

## 8 Discussion

This present study aimed to understand the practices of health professionals in the hospital setting, and affiliated care settings, when treating children and young people who present following self‐harm. Previous explorations of emergency department interactions for self‐harm have centred on negative professional attitudes and at times punitive treatment (Chandler 2016, Saunders *et al*. 2012), but there has been limited interrogation of the rationales behind these attitudes and the factors that inform professionals’ approach. Drawing on systems theorising within sociology, we are able to draw out the interactions between system actors and explore what is considered meaningful within system structures (Luhmann 1986, Parsons 1951). Overall, a systems perspective emphasises the importance of dynamics, relationships and interactions among different system elements and sub‐systems, which may have previously been overlooked (Hawe *et al*. 2009).

In this study, a complex systems perspective provided a means for: understanding professional attitudes in relation to wider systems; unpacking the contextually contingent nature of interactions; and highlighting both the potential and limits of individuals’ agency for enacting change within the system. Professionals conceptualised self‐harm within a framework of risk management, and the adoption of a complex systems lens provided explorations of how risk was assessed alongside priorities and values from a range of sub‐systems, particularly biomedical discourses and constructions of health and illness. This confirms developments within the sociology of risk which recognise the complexity of decision‐making and the influence of political, cultural and societal sub‐systems (Alaszewski 2009, Green 2009). Conceiving a hospital emergency department through a complex systems lens, reminds us about the importance of nested systems, consisting of a range of agents within their own sub‐systems and within a wider structure shaped by the local healthcare system (Keshavarz *et al*. 2010). Strongly held views about professional role boundaries and keeping ‘within a medical box’ resonate with the dominance of biomedical sub‐systems in managing self‐harm (Chandler 2016) and the way in which medical models separate the treatment of bodies and minds (Hadfield *et al*. 2009). As this and others studies have indicated, professionals’ defence of role boundaries is often driven by feelings of fear, anxiety and uncertainty in relation to self‐harm presentations, and explorations of patient responses would help further understand the influence of these medical sub‐systems (O'Connor and Glover 2016).

Accounts also illustrated the importance of moving patients through the care pathway, which meant that professionals were not always positively orientated to the patient, as their priority was to shift them forward. This resonates with findings elsewhere about the temporal constraints placed on health service delivery where systemic aims to reduce service‐provision costs and increase rates of discharge so as to treat more people overall, overlook the complexity of what is involved in patient care (McWade 2015).

Frustrations about progressing patients were intertwined with accounts of bounded and discrete expertise, with professionals defending their area of specialism and ‘retreating towards the protected core’ (Larson 1990: 45). In this study, we see these dynamics played out as professionals sometimes distance themselves from self‐harm as an area which is outside their own specialism, while also grappling with where expertise is located along the care pathway. This sort of boundary work confirms what other studies have reported about how professionals categorise patients as unsuitable for certain types of care before re‐directing to other, more appropriate care contexts – a form of medical gatekeeping which is facilitative rather than restrictive (Buchbinder 2017). As well as facilitating patient access to more appropriate care, moving patients out of the emergency department also reduces strains on resources thereby responding to system demands and institutional pressure points (Hillman 2014).

Exploring the underlying factors behind professional practices highlighted detrimental implications for the quality of patient care. Assessing individuals in relation to risk rather than ‘illness’ meant that children and young people were sometimes seen as ineligible for medical treatment, and the culture of ‘moving on’ led to fleeting interactions and limited relationship building. As O'Connor and Glover (2016: 494) comment, these sorts of superficial patient encounters result from ‘prioritising systemic rather than patient needs’. Uncertainty over medical expertise impacted on professionals’ confidence in handling self‐harm presentations and also meant that patients were often left without information, support or advice. These implications resonate with previous findings about negative patient experiences of emergency care, but having unpacked the determinants of professional practices, we can better interpret these experiences within the context of system challenges and drivers.

To attempt to counterbalance the system‐level drivers that were considered to contribute to cases of inadequate care, a small number of professionals described going beyond their role and exerting agency to enact institutional change. These accounts of agency to change were mainly given by nursing staff, reflecting other reports about less negative attitudes held by nurses towards patients who self‐harm (Saunders *et al*. 2012). Highlighting these individual responses to structural inadequacies reminds us how patient experiences are not entirely shaped by large‐scale systems and structures (Buchbinder 2017).

However, ultimately this individual‐level effort was limited, and often futile in the contexts of entrenched systems structures. Indeed, efforts to improve care quality were restricted by spatial, temporal and financial challenges to these changes. This illustrates that there were small‐scale efforts by a limited number of individuals in the system to change, and in some cases it is an active process, but the current barriers are insurmountable within a coordinated, whole system approach.

### 8.1 Study limitations

There are three key limitations of the study’s methods. First, the findings are highly contextualised as the study was based on a single case study hospital (Crowe *et al*. 2011). Second, while we mapped the system to identify a complete set of stakeholders, missing were perspectives from adult emergency department clinical staff (although one adult emergency department nurse participated) and also the emergency department reception team (the first point of contact). Third, the study includes interview data rather than ethnographic, observational data, which does not permit us to understand interactions with system structures in situ. However, the study offers an important direction of travel for generating theory on system‐level influences on medical professional practices.

### 8.2 Implications

The present study provides important implications for research, policy and professional practice in regard to the management and prevention of self‐harm. First and foremost, we need more idiographic, ethnographic research to provide further insights into the dynamic interactions between individuals and systems structures in situ, examining health professionals’ agency to change, and also extending our study to more diverse hospital settings (Allen 2015, Bantjes *et al*. 2017, Van Keer *et al*. 2017). Secondly, future research should investigate how health professionals differentiate between types of patients, including first time and frequent attenders. To date, there has been a tendency to view self‐harm patients as a homogenous group (MacDonald *et al*. 2020) and unpacking nuance between different sorts of patients is a clear research priority. Finally, there is also scope to look beyond the sites where professional–patient interactions occur, and consider in more detail the precipitating factors that contribute to our observations of practice. For example, research might attend more closely to professional training and its assumptions of medicine as a set of discrete specialisms (Atkinson 1984, Gale 2009). This would provide further insights into the place of self‐harm within this system of specialisation and how it might evade being the responsibility of any particular professional group.

In regard to policy and practice, one of the central areas for work is to improve the culture about who assumes responsibility for self‐harm. In this sense, there is the potential to de‐mystify the notion that its management can only fall within the purview of a small number of experts, and instead promote the idea that basic skills may be enacted by all professionals. Equally however, there does need to be more awareness of the diverse roles within hospital settings and where additional support may be drawn. Professionals often constructed their identity in relation to others – but these other roles were often poorly defined or understood.

Furthermore, there is an evident need for improved processes for taking a systemic approach when enhancing the quality of care provision across the whole pathway for self‐harm as presently change appears over reliant on the actions of a small number of motivated individuals. For many, this systemic change needs to be orientated towards the ‘boring’ or general aspects of system infrastructures (Greenhalgh *et al*. 2019), ranging from provision of appropriate space, clearer guidelines for post‐discharge care and increased opportunities for supervisory support. Current guidance for the management and prevention of self‐harm could be revisited to improve the multidisciplinary approach and enhance patient experiences (Courtney *et al*. 2019, National Institute for Health and Care Excellence 2004). Variable implementation of clinical guidance suggests the importance of structural change to support guidance delivery and addressing resource limitations is an obvious place to start.

## 9 Conclusion

Adopting a systems approach to understanding the prevention and management of self‐harm, provides a nuanced understanding of the complex contextual factors that structure professionals’ practices. The study revealed that hospitals are characterised by a culture of risk management and patient progress, which detrimentally impacts the quality of care professionals feel able to provide. These issues are exacerbated by challenges in possessing or locating expertise in treating self‐harm within the system. While there is evidence of small‐scale innovation and efforts to instigate change at the individual level, there remain limited mechanisms to scale these up or enact structural‐level change.

## Declaration of competing interests

The authors declare no potential conflict of interest with respect to the research, authorship and/or publication of this article.

## Author Contributions

**Sarah MacDonald:** Conceptualization (supporting); formal analysis (lead); investigation (supporting); methodology (equal); project administration (supporting); validation (equal); visualization (lead); writing – original draft (lead); writing – review and editing (equal). **Catherine Sampson:** Conceptualization (supporting); formal analysis (supporting); investigation (lead); methodology (equal); project administration (supporting); supervision (supporting); validation (equal); visualization (supporting); writing – original draft (supporting); writing – review and editing (equal). **Lucy Biddle:** Conceptualization (supporting); funding acquisition (supporting); investigation (supporting); methodology (supporting); writing – review and editing (supporting). **Sung Yeon Kwak:** Formal analysis (supporting); validation (supporting); writing – original draft (supporting); writing – review and editing (supporting). **Jonathan Scourfield:** Conceptualization (supporting); funding acquisition (supporting); supervision (supporting); writing – review and editing (supporting). **Rhiannon Evans:** Conceptualization (lead); formal analysis (equal); funding acquisition (lead); investigation (lead); methodology (equal); project administration (lead); supervision (lead); validation (supporting); writing – original draft (supporting); writing – review and editing (equal).

## Funding

The authors disclose receipt of the following financial support for the research, authorship and/or publication of this article: The study was funded by Health and Care Research Wales (Project Reference 1319). The views expressed in this publication are those of the author(s) and not necessarily those of Health and Care Research Wales. The work was undertaken with the support of the Centre for the Development and Evaluation of Complex Interventions for Public Health Improvement (DECIPHer), a U.K. Clinical Research Collaboration (UKCRC) Public Health Research Centers of Excellence. Joint funding (MR/KO232331/1) from the British Heart Foundation, Cancer Research UK, Economic and Social Research Council, Medical Research Council, the Welsh Government, and the Wellcome Trust, under the auspices of the U.K. Clinical Research Collaboration, is gratefully acknowledged.

## Data Availability

The data that support the findings of this study are available on request from the corresponding author. The data are not publicly available due to privacy or ethical restrictions.
